# A survey among physicians in surgery and anesthesiology departments after the first surge of SARS-CoV-2 infections in Germany: Preparing for further challenges ahead

**DOI:** 10.1007/s00508-021-02000-z

**Published:** 2022-01-21

**Authors:** Anna Grishina, Fabian Link, Arne Arend, Florentine Kleemann, Pinkus Tober-Lau, Dominik Andree, Friederike Münn, Magdalena Gruendl, Markus Quante, Hans Lederhuber, Markus Albertsmeier, Florian Struller, Robert Grützmann, Alfred Königsrainer, Markus W. Löffler

**Affiliations:** 1 Department of Pediatrics I, University Medicine Essen, University of Duisburg-Essen, Essen, Germany; 2 University Hospital Halle (Saale), Halle, Germany; 3 Martin-Luther-University Halle (Saale), Halle, Germany; 4 Medical Faculty, Greifswald University Hospital, Greifswald, Germany; 5 Department of Infectious Diseases and Respiratory Medicine, Charité-Universitätsmedizin Berlin, Berlin, Germany; 6 Faculty of Arts and Social Sciences, Open University, Milton Keynes, UK; 7 Department of Sport and Health Sciences, Chair of Epidemiology, Technical University Munich, Munich, Germany; 8 Department of General, Visceral and Transplant Surgery, University Hospital Tübingen, Tübingen, Germany Hoppe-Seyler-Str. 3, 72076; 9 Department of Colorectal Surgery, Royal Devon and Exeter NHS Foundation Trust, Exeter, UK; 10 Department of General, Visceral and Transplantation Surgery, LMU University Hospital, Ludwig-Maximilians-University (LMU) Munich, Munich, Germany; 11 Department of Surgery, University Hospital Erlangen, Erlangen, Germany; 12 Cluster of Excellence iFIT (EXC2180) “Image-Guided and Functionally Instructed Tumor Therapies”, University of Tübingen, Tübingen, Germany; 13 Department of Immunology, Interfaculty Institute for Cell Biology, University of Tübingen, Tübingen, Germany; 14 Department of Clinical Pharmacology, University Hospital Tübingen, Tübingen, Germany

**Keywords:** COVID-19, Healthcare, Stress factors, Working conditions, Work-related dissatisfaction

## Abstract

**Background:**

The SARS-CoV‑2 pandemic has extensively challenged healthcare systems all over the world. Many elective operations were postponed or cancelled, changing priorities and workflows in surgery departments.

**Aims:**

The primary aim of this cross-sectional study was to assess the workload and psychosocial burden of surgeons and anesthesiologists, working in German hospitals during the first wave of SARS-CoV‑2 infections in 2020.

**Methods:**

Quantitative online survey on the workplace situation including psychosocial and work-related stress factors among resident and board-certified surgeons and anesthesiologists. Physicians in German hospitals across all levels of healthcare were contacted via departments, professional associations and social media posts.

**Results:**

Among 154 total study participants, 54% of respondents stated a lack of personal protective equipment in their own wards and 56% reported increased staff shortages since the onset of the pandemic. While routine practice was reported as fully resumed in 71% of surgery departments at the time of the survey, work-related dissatisfaction among responding surgeons and anesthesiologists increased from 24% before the pandemic to 36% after the first wave of infections. As a countermeasure, 94% of participants deemed the establishment of action plans to increase pandemic preparedness and strengthening German public health systems a useful measure to respond to current challenges.

**Conclusion:**

The aftermath of the first wave of SARS-CoV‑2 infections in Germany has left the surgical staff strained, despite temporarily decreased workloads. Overall, a critical review of the altered conditions is indispensable to identify and promote effective solutions and prudent action plans required to address imminent challenges.

## Introduction

The ongoing severe acute respiratory syndrome coronavirus type 2 (SARS-CoV‑2) pandemic has directly impacted the situation of healthcare professionals and has caused alarming effects on a global scale [1–3]. In addition to high workloads and limited resources, healthcare professionals are now facing various unprecedented challenges. Besides a relevantly increased risk of becoming infected and falling ill themselves [4–6], the pandemic has also had a considerable impact on the workload of healthcare providers [7–9]. In particular, the daily routines in surgical departments have massively changed during the pandemic. Since patients contracting SARS-CoV‑2 during or after a surgical intervention are at risk of developing severe perioperative complications and have high mortality rates [10], elective surgical procedures were postponed or cancelled in many hospitals, intending to protect patients [10–12]. Such measures also aimed at preserving sufficient capacities for critically ill coronavirus disease 2019 (COVID-19) patients at intensive care units. As a result, numerous surgical procedures that have been postponed will have to be performed between COVID-19 waves or later on [13]. After infection surges, this will likely result in increasing workloads for surgical departments without the possibility of compensating these challenges by additional staff [14].

The primary objective of this study was therefore to assess the changes regarding workload and psychosocial burden for surgeons and anesthesiologists in the aftermath of the first wave of SARS-CoV‑2 infections in Germany. In addition, the survey assessed participants’ resilience and mitigation strategies with a focus on potential solutions for future surges and challenges, referring to the general health system level in Germany.

## Material and methods

**Fig. 1:**
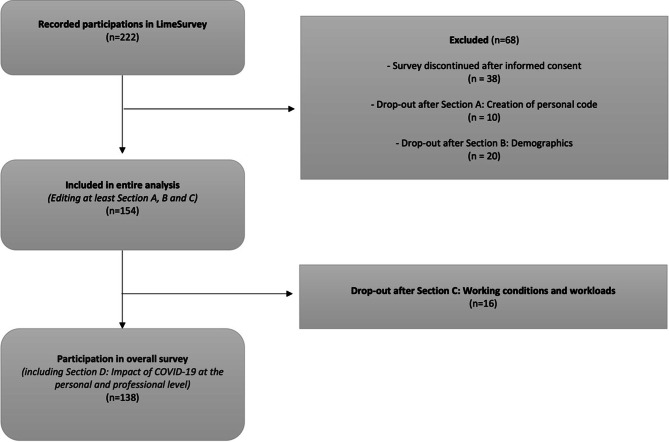
Flow chart of study participants. The flow chart depicts the participation in the different survey sections via the LimeSurvey platform with respective numbers (*n*). Since several participants dropped out, the number (*n*) of participants in each section is presented, alongside the drop-out rates by section. Overall, 138 people completed the entire survey

A self-developed web-based questionnaire was created using the online survey platform LimeSurvey (LimeSurvey GmbH, Hamburg, Germany; https://www.limesurvey.org). Invitations to participate in the survey with an access link were sent by mail to the official contacts of surgery and anesthesiology departments in over 300 hospitals across all healthcare levels in Germany with a request to forward them to respective department members, as well as communicated through professional associations and posts on social media channels. The questionnaire was created exclusively for this study. Survey participation was possible from 19 July until 30 September 2020. Participants were physicians working across all surgical disciplines (including e.g., general surgery, ophthalmology and gynecology) or anesthesiology departments in German hospitals (employed since 1 January 2020 or before). Participants were contacted through their institutional email and by means of different associations, thus a concrete sample size regarding persons inquired for survey participation cannot be provided. Participants responding to the questionnaire gave their consent before starting the survey, including the use of their anonymized data. The study protocol was reviewed by the local Ethics Committee at the University of Tübingen and a positive vote was obtained (project number: 513/2020BO). The survey consisted of 49 questions (in German). One section of the survey collected demographic data (including sex, age, ethnicity, marital status, etc.), another addressed working conditions and workload before the pandemic and own health-related risk factors as well as information on continuation of elective surgical procedures. The last section of the survey focused on the identification of psychosocial stress factors due to the SARS-CoV‑2 pandemic, covering both the personal as well as work-related settings (Fig. 1). The questionnaire comprised a mixture of question styles, including dichotomous questions (yes/no questions), single selection questions, multiple choice questions and Likert-type scale items (including answer formats such as: strongly agree/agree/neither agree nor disagree/disagree/strongly disagree).

**Fig. 2:**
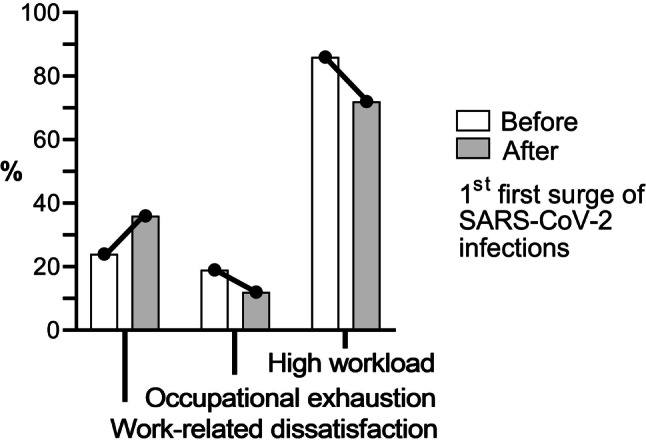
Changes in working conditions comparing the situation before and during the pandemic. The bar chart depicts % changes in work-related aspects before and during the pandemic (after the first wave of SARS-CoV‑2 infections in Germany) as reported by survey participants (*n* = 138). The items presented are shortened statements from original questions. An exemplary pair of items (as authors’ translation from German) used is *“*Before the onset of the COVID-19 pandemic, my workload was high” and “Currently, my workload is high”

**Fig. 3:**
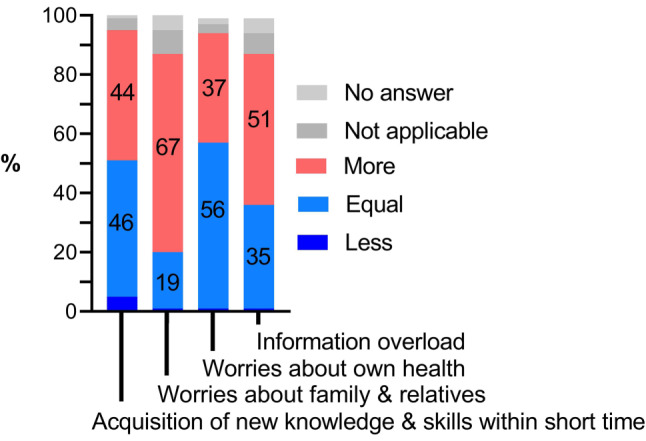
Work-related stress factors perceived by study participants. The bar graph illustrates % fractions of selected work-related stress factors during the SARS-CoV‑2 as increased (more), equal or less, when compared to the situation before the pandemic by survey participants (*n* = 138)

To assess the workload, work-related satisfaction as well as occupational exhaustion before and during the SARS-CoV‑2 pandemic, two different time frames in consecutive order with structurally identical items were provided. Questions on subjective perceptions, addressing aspects such as workload or job satisfaction before and during the SARS-CoV‑2 pandemic were assessed as consecutive questions using 5‑point Likert-type scale items with the additional answer option “No answer”, ranging from “Strongly disagree” to “Strongly agree”. Using a 11-item section of the questionnaire, the first six items inquired to what extent the respondents agreed or disagreed with statements concerning their personal workload, work satisfaction and occupational exhaustion (Fig. 2). In the following, several examples for the phrasing of typical questions are provided, as translated by the authors from the German original. A first block of questions was used to evaluate the situation in retrospect (e.g.: “Before the onset of the COVID-19 pandemic, my workload was high”, “Before the onset of the COVID-19 pandemic, I felt overwhelmed at work” or “Before the onset of the COVID-19 pandemic, I often felt dissatisfied at work”). Subsequently, to evaluate the current situation statements like “Currently my workload is high”, “Currently I feel overwhelmed at work” or “Currently, I often feel dissatisfied at work” were inquired in a second block of questions belonging to the same set of items. To evaluate which factors influence work-related dissatisfaction, the participants were asked to what extent several stress factors changed during the pandemic, on a 5-point Likert scale ranging from “Strongly decreased” to “Strongly increased” complemented by the two further answer options “Not applicable” and “No answer”. An exemplary item for this category of questions is “Acquisition of new knowledge and skills within a short time has …” (Fig. 3).

### Statistical analysis

Data was analyzed using IBM SPSS Statistics version 26.0 (IBM Corp., Armonk, NY, USA). Data are presented as total numbers (*n*) and means. To estimate the statistical association between different questions on the questionnaire, Spearman’s rank correlation coefficient was used. To prevent the accumulation of alpha errors, a Bonferroni correction was performed. We assumed *p* < 0.05 to indicate statistically significant differences.

## Results

### Demographics

Among 222 started surveys recorded in LimeSurvey, 154 participants completed the survey at least in parts and were included in the analysis. Sixty-eight survey responses were excluded from the analysis due to discontinuation before completing the relevant sections B and C. In total, 138/154 participants (90%) completed the full survey (Fig. 1). From the full study cohort 58/154 participants (38%) indicated to be females and 94/154 (61%) to be males (in two cases data were lacking). Age distribution is shown in Table 1. Out of the 154 anesthesiologists and surgeons, 54 identified as residents (35%) and 97 as board-certified physicians (63%, in three cases data were lacking). Thereof, 40/97 (41%) stated that they work as a head of department (Table 1). Respondents were from hospitals all over Germany, including all levels of healthcare, with a predominant share from maximum care and university hospitals (57%). Further information, including data related to levels of hospital care and location, is provided in Table 1. The subdivision of the cohort according to specialization is provided in Table 2.

**Table 1:** Study participant characterization (*n* = 154) and hospital information

		*n*	%
**Sex**			
Female		58	38
Male		94	61
No answer		2	1
**Age (years)**			
25–34		54	35
35–44		35	23
45–54		40	26
55–64		23	15
≥ 65		1	< 1
No answer		1	< 1
**Professional status**			
Resident		54	35
Specialist physician (board certified)		20	13
Consultant (senior physician)		37	24
Chief physician (head of department)		40	26
No answer		3	2
**Hospital category**			
*First-level hospital care*	Basic provider hospital	18	12
	Outpatient surgery center	1	< 1
*Second-level hospital care*	Focus provider hospital	46	30
*Third-level hospital care*	Maximum care hospital	21	14
	University hospital	67	43
No answer		1	< 1
**Location of hospitals**			
North Rhine-Westphalia		35	23
Baden-Württemberg		32	21
Bavaria		20	13
Lower Saxony		15	10
Hesse		10	7
Saxony		7	5
Berlin		6	4
Rhineland-Palatinate		5	3
Saxony-Anhalt		5	3
Schleswig-Holstein		5	3
Thuringia		4	3
Brandenburg		3	2
Bremen		2	1
Hamburg		2	1
Saarland		2	1
No answer		1	< 1

**Table 2:** Classification of study participants by specialization and level of professional education (*n* = 154)

Specialization	*n*	%
*Residents (n* *=* *54)*		
General surgery	4	7
Anesthesiology	15	28
Ophthalmology	2	4
Dermatology	1	2
Vascular surgery	1	2
Gynecology	1	2
Otorhinolaryngology	2	4
Pediatric surgery	1	2
Oral and maxillofacial surgery	2	4
Neurosurgery	1	2
Orthopedics and trauma surgery	2	4
Plastic and aesthetic surgery	2	4
Urology	2	4
Visceral surgery	18	33
*Board-certified physicians (n* *=* *97)*		
General surgery	21	22
Anesthesiology	28	29
Ophthalmology	3	3
Dermatology	1	1
Vascular surgery	5	5
Gynecology	4	4
Otorhinolaryngology	3	3
Cardiac surgery	1	1
Oral and maxillofacial surgery	2	2
Neurosurgery	3	3
Orthopedics and trauma surgery	8	8
Thoracic surgery	5	5
Urology	2	2
Visceral surgery	11	11
*Unknown (n* *=* *3)*	–	–

### Workload and psychosocial burden

From July to September 2020, 83/154 participants (54%) reported to have experienced shortages of personal protective equipment (PPE) at their department, of whom 46% (38/83) specified that these periods exceeded 4 weeks. Shortages in staffing since the onset of the SARS-CoV‑2 pandemic were reported by 86/154 (56%) respondents. About half of the participants (80/154; 52%) reported sick leave rates at less than 10%, 34/154 (22%) at 10–20% and 11/154 (7%) at 20–30%, whereas 3 participants (2%) reported substantially elevated rates at 30–40%. A sick leave rate of 0% was only reported by 4 participants (3%). Twenty-two participants (14%) did not answered the question. Shortages concerning drug supplies were mentioned by 48/154 (31%) participants, as well as shortages in sterile goods (e.g., surgical instruments, wound care supplies, surgical gloves) by 50/154 (32%) of participants.

Compared to routine practice before the pandemic, 47/154 respondents (30%) reported estimated the decrease of elective procedures at 60–80% between 1 March 2020 and 1 June 2020, and 25% (38/154) estimated the decrease at 40–60% for elective procedures during this period. At the time of the survey (July to September 2020), most participants (110/154; 71%) reported that their hospital had resumed routine surgical practice, also including intensive care units. Seven respondents (5%) alluded to an outbreak of SARS-CoV‑2 after resuming elective procedures at their surgical department (Table 3).

**Table 3:** Survey replies concerning the resumption of routine practice and elective surgery (*n* = 154)

	(*n*)	%
*Resumed routine practice* (including intensive care units)		
Yes	110	71
No	38	25
“I don’t know”	6	4
*Rate of postponed elective procedures (1 March–1 June 2020)*		
0–≤ 20%	15	10
> 20–≤ 40%	23	15
> 40–≤ 60%	38	25
> 60–≤ 80%	47	30
> 80–≤ 100%	19	12
“I don’t know”	12	8
*Time period before resuming elective surgery*		
Since the outbreak of the pandemic, elective surgery has been performed in our department without restrictions	2	1
< 3 weeks	1	< 1
3–6 weeks	46	30
7–10 weeks	60	39
> 10 weeks	38	25
No elective surgeries are currently performed	1	< 1
No answer	1	< 1
Other^a^	5	3
*Did you have a SARS-CoV‑2 outbreak at your surgical ward after resuming elective surgeries?*		
Yes	7	5
No answer	8	5

^a^Reduced elective program since April 2020 until today; elective oncological surgery was continued, the surgical capacity is still limited

Work-related dissatisfaction was reported to exist by 24% of all participants (33/138) already before the onset of the pandemic, which increased to 36% (50/138) after the first wave of infections in Germany. In more detail, work-related dissatisfaction was reported by 30% (14/47) of responding residents and 21% (19/91) of board-certified surgeons and anesthesiologists as prevalent already before the pandemic, increasing to 40% (19/47) among responding residents and to 34% (31/91) for board certified surgeons and anesthesiologists, following the first wave. The workload before the SARS-CoV‑2 pandemic was rated as high by 86% (119/138) of participants, whereas only 72% (99/138) still rated their personal workload as high at the time of the survey. A daily workload rated as excessive was reported by 19% (26/138) of participants before the pandemic, which decreased to 12% (16/138) at the time of the survey (Fig. 2).

Concerning stress factors potentially impacting professional dissatisfaction, a perception of threat by COVID-19 was indicated by 54/138 participants (39%). A deterioration in patient care as an additional relevant factor was mentioned by 63/138 participants (46%). Other factors potentially associated with increased professional dissatisfaction are provided in Table 4.

**Table 4:** Survey replies concerning associated factors for work-related dissatisfaction (*n* = 138)

	Strongly disagree	Somewhat disagree	Neither	Somewhat agree	Strongly agree	No answer
*I feel threatened by the COVID-19 pandemic*	26	33	23	48	6	2
	(19%)	(24%)	(17%)	(35%)	(4%)	(1%)
*Patient care has deteriorated due to the COVID 19 pandemic*	14	36	23	39	24	2
	(10%)	(26%)	(17%)	(28%)	(17%)	(1%)
*I feel comfortable caring for patients despite the current situation*	2	13	10	68	44	1
	(1%)	(10%)	(7%)	(49%)	(32%)	(< 1%)
*I have an extensive knowledge of COVID-19, so I am able to provide adequate care for patients*	5	21	32	60	18	2
	(4%)	(15%)	(23%)	(43%)	(13%)	(1%)
*Because elective surgeries are cancelled or postponed during the pandemic my workload has increased*	7	24	22	56	27	2
	(5%)	(17%)	(16%)	(41%)	(20%)	(1%)
*The COVID-19 pandemic has caused traumatic events in my professional work*	70	37	18	8	3	2
	(51%)	(27%)	(13%)	(6%)	(2%)	(1%)
*There are now more conflicts with colleagues and/or superiors*	27	47	30	21	13	–
	(20%)	(34%)	(22%)	(15%)	(9%)	

Further common stress factors related to the pandemic included increased worries about family and relatives 92/138 (67%), worries about the own health status 51/138 (37%), information overload 71/138 (51%) and the requirement for acquisition of new knowledge and skills within a short time 60/138 (44%) (Fig. 3).

**Fig. 4:**
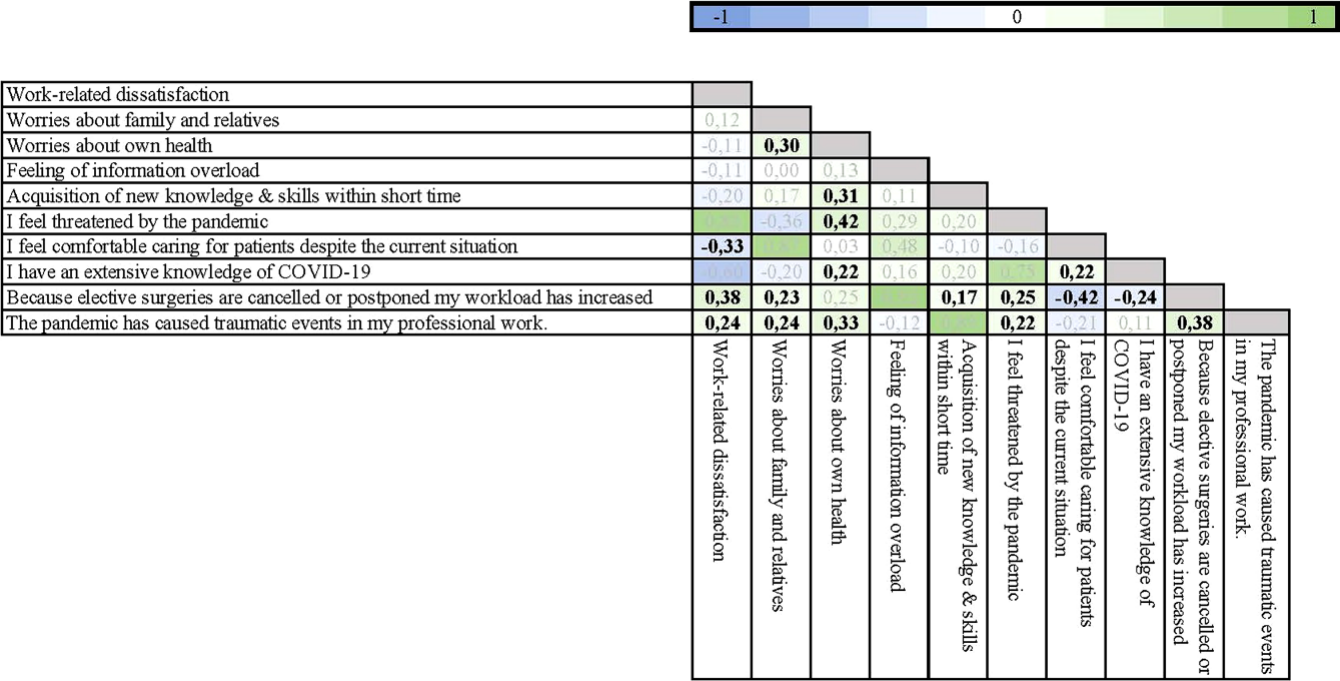
Correlation analysis of survey replies concerning different stress factors. The correlation matrix displays the statistical relation of answers on the questionnaire as Spearman’s rank correlation coefficient. Statistically significant results are printed in bold, non-significant results are grayed out. A correlation coefficient is usually considered to indicate a negligible correlation from 0.00 to ±0.19, a weak correlation from ±0.20 to ±0.39, a moderate correlation from ±0.40 to ±0.59, a strong correlation from ±0.60 to ±0.79, and a very strong correlation from ±0.80 to ±1.00. A positive correlation coefficient indicates a positive relationship between the two variables, while a negative correlation coefficient indicates a negative relationship

To analyze whether answers on the questionnaire were correlated, Spearman’s rank correlation coefficient ρ was calculated for the ordinally scaled datasets. Here it was found that a sense of high workload and traumatic events due to the pandemic were associated with the most negative perceptions regarding work (Fig. 4).

The indication of work overload was significantly correlated with work dissatisfaction (ρ = 0.38), worries for relatives (ρ = 0.23), feeling the necessity to learn many new things in a short period of time (ρ = 0.17) as well as feeling threatened by the pandemic (ρ = 0.25). On the other hand, there was a significant negative correlation with the report of work overload and the feeling of providing good care to patients (ρ = −0.42) and feeling well-informed about COVID-19 (ρ = −0.24). Reporting traumatic events due to the pandemic was significantly correlated with work dissatisfaction (ρ = 0.24) and work overload (ρ = 0.38), worries for relatives (ρ = 0.24) and for one’s own health (ρ = 0.33) as well as the perception of being threatened by the pandemic (ρ = 0.22). Although all of these findings were formally significant, it should be duly noted that single correlations remained weak to moderate throughout and therefore require context for sensible interpretation.

### Mitigation strategies for future challenges

For an improved management of prevalent shortfalls caused by the pandemic as well as the encountered challenges, various predefined measures were to be rated as potentially useful by the survey participants. Most respondents (129/138; 94%) agreed that action plans to increase pandemic preparedness and for strengthening public health systems are useful and important countermeasures. Also, additional research into novel approaches to increase protection of healthcare workers from SARS-CoV-2-related health risks was mentioned as a sensible measure by the vast majority of participants (127/138; 92%). According to most participants in the survey, national (127/138; 92%) as well as regional (118/138; 85%) guidelines to counteract an outbreak in the context of epidemic infectious diseases should be established. Establishment of concepts for more efficient staff deployment during crises was also rated as useful by 115/138 (83%) of the respondents. Strengthening interprofessional cooperation and communication was frequently deemed as a helpful measure (80%) as well as prioritized diagnostic testing for healthcare personnel during a pandemic (83%), whereas a hastened return to prepandemic conditions was rejected by 59% of survey respondents (Table 5).

**Table 5:** Survey replies concerning the evaluation of mitigation strategies (*n* = 138)

	Not useful	Somewhat useful	Useful	No answer
Return to prepandemic conditions	81	26	28	3
	(59%)	(19%)	(20%)	(2%)
Establishment of action plans to increase pandemic preparedness and to strengthen public health systems	–	6	129	3
		(4%)	(94%)	(2%)
Research of new approaches to better protect medical and nursing staff from risks in pandemics	2	8	127	1
	(1%)	(6%)	(92%)	(< 1%)
Preparation of national guidelines for action to combat the pandemic	2	8	127	1
	(1%)	(6%)	(92%)	(< 1%)
Preparation of regional and local action plans to combat the pandemic	7	12	118	1
	(5%)	(9%)	(85%)	(< 1%)
Establishment of concepts for more efficient personnel deployment in times of crisis	6	15	115	2
	(4%)	(11%)	(83%)	(1%)
Extension of inter-professional cooperation and communication with other fields	5	20	111	2
	(4%)	(15%)	(80%)	(1%)
Prioritized testing of healthcare workers in the case of a pandemic	6	17	114	1
	(4%)	(12%)	(83%)	(< 1%)

## Discussion

The aftermath of the first and subsequent waves of SARS-CoV‑2 infections has burdened the surgical and anesthesiologic staff in Germany. Our survey attests that elective surgery was relevantly impacted by postponed or cancelled procedures to minimize the risk of perioperative SARS-CoV‑2 infection and to free up healthcare resources. A key challenge of the pandemic were staff shortages, as frequently mentioned by participating physicians. Furthermore, stress perceived through impaired patient care and/or the feeling of anxiety by the SARS-CoV‑2 pandemic may be associated with the increased work-related dissatisfaction identified. Increased worries about family and relatives may be considered another main stress factor, adding to the psychosocial burden of healthcare staff. It seems important to review issues that have been encountered during the initial phase of the pandemic to enable the development of action plans for increasing pandemic preparedness and supporting public health systems.

The answers provided to our survey confirmed shortages in basic supplies such as the availability of PPE. Most surgeons and anesthesiologists reported witnessing a shortage of PPE within their department after the onset of the pandemic. Comparable results were reported by Brunner et al. who reported a lack of PPE in 45% of 101 participating German hospitals [15]. Furthermore, a survey conducted by the Royal College of Surgeons of England among 1978 surgeons revealed that one third of them lacked access to adequate PPE [16]. In a global online survey by Tabah et al. 52% of respondents indicated a lack of adequate access to at least one PPE item [17]. According to our survey, 54% of the participants experienced a lack of PPE, among whom 46% reported this shortage to last for at least 4 weeks. In an international study from 26 countries by Yanez Benitez et al. 54% of surgeons responded that their work was impaired by the lack of PPE and 51% stated that they did not feel sufficiently protected by the PPE provided [18].

According to our survey, drug supply shortages were indicated by 31% and shortages in sterile goods by 32% of respondents. This circumstance appears critical for patient care, also when the strongly increased demand for certain essential drugs (e.g. sedatives) is taken into account [19]. Studies concerning shortages of staff and supplies were unavailable at the time of our study, leaving the final assessment of the international situation still open; however, more recently the COVIDSurg Collaborative has provided an international surgical workforce prediction model, addressing COVID-19 related absences in the surgical setting. During the initial 6 weeks of the global outbreak the COVID-19-related absence of surgeons ranged between 20.5% and 24.7%, which corresponds well with the data from our survey [20].

Postponing and cancelling elective surgery was introduced in Germany on 16 March 2020 and has lasted to varying degrees over the following year, in order to reserve capacities on intensive care units for COVID-19 patients and to reduce the perioperative risk for patients [13]. The restrictions in surgical capacity were substantial, especially during the first national lockdown lasting from 1 March to 1 June 2020. Our survey supports this, as 55% of respondents estimated the cancellation rate of elective surgery to range between 40% and 80% and 12% of respondents even estimated this to be higher than 80%. This picture fits the international situation at the time, as, e.g. an international study in 54 countries reported a COVID-19-related overall reduction of oral and maxillofacial surgery activities by 56% [21]. Respondents to a study from Johnson et al. alluded to a decrease of 90% for vascular surgeries in the USA [22] and in Austria, a complete cancellation of elective vascular surgery was reported by most of the 12 centers surveyed during the first surge of the pandemic [23]. Of note, 5% of respondents to our survey stated that they had become aware of a SARS-CoV‑2 outbreak at their department after resuming elective procedures, leading to repeated cancellations of elective procedures. Anecdotally, such a phenomenon has been reported before, e.g. in a study from Wuhan, China, where two SARS-CoV‑2 outbreaks were reported on a surgical ward involving both patients and healthcare workers [24], or likewise in a report from a hemodialysis unit in Canada [25]. A premature return to regular elective surgery without appropriately adapted mitigation measures therefore involves a relevant risk of infection outbreaks. To lower the risk of nosocomial infections, COVID-19-free pathways for elective surgery have been proposed and were linked to a positive impact when aiming to protect vulnerable patients [26] as well as vaccinations before elective surgery [27]. Systematic PCR testing of patients before surgical procedures has also been proven as a useful tool for preventing COVID-19-related complications [28].

The SARS-CoV‑2 pandemic has also relevantly affected the personal lives of physicians. According to the survey data, most respondents stated that worries concerning family and relatives have significantly increased, contributing additional stressors. This notion is supported by a study from Collins et al. where worries for immediate family members were identified as one of the main stress factors during the current pandemic [29]. Furthermore, Tan et al. [30] proposed that longitudinal mental support should be made accessible to healthcare workers in exceptional situations, such as a pandemic, a view which may generally be supported by our results attesting to a negative impact of (traumatic) events during the pandemic on work-life balance. For future public health crises, this could be considered when developing measures to support healthcare workers. Immediate measures might therefore also include close family of healthcare professionals.

The presented study findings are limited by the small sample size, which may reduce the generalizability and question the validity of findings across Germany and different hospital settings. Another limitation is a primary study focus on the subjectively perceived stress factors among the study participants as well as asking for events in the past. Due to this fact, recall bias as a potential methodological issue influencing the results cannot be excluded. This survey, however, was not planned as a longitudinal study with repeat questionnaires, therefore such changes cannot be addressed over time.

This present study is one of the very few studies conducted in Germany that comprehensively addressed psychosocial stressors as well as mitigation measures after the first peak of the SARS-CoV‑2 pandemic. A major strength of our study is the coverage of various challenges at different levels (occupational, personal, co-environmental). Furthermore, besides the assessment of psychosocial burden, measures at the level of the public health system were queried, representing a prerequisite to identify relevant problem areas when searching for potential solutions.
